# Study Suggests Long-Term Decline in French Sperm Quality

**DOI:** 10.1289/ehp.121-a46

**Published:** 2013-02-01

**Authors:** Adrian Burton

**Affiliations:** Adrian Burton is a biologist living in Spain who also writes regularly for *The Lancet Oncology*, *The Lancet Neurology*, and *Frontiers in Ecology and the Environment*.

New study findings suggest widespread declines in sperm quality in French men between 1989 and 2005, with average sperm counts falling while percentages of abnormally formed sperm rose. These findings are a “serious public health warning,” the authors wrote, although they point out the average estimated sperm count is still well above the level deemed normal by the World Health Organization.^1^

Recent years have seen many similar reports of falling human sperm counts, but there has been much debate over whether the problem is real. “The principal trouble has been selection bias,” says Joëlle Le Moal, an environmental health epidemiologist at the Institut de Veille Sanitaire in Saint Maurice, France, and joint first author of the new article with colleague Matthieu Rolland. She explains that few studies have involved sperm samples collected from randomly selected members of the general population; for the few that did, only a small percentage of men agreed to be included, and the studies were run in restricted areas. So most studies have had to rely on sperm donors or couples coming to fertility clinics, which do not represent the general population.

However, although this study population also involved couples attending fertility clinics, the men were partners of women known to be totally infertile—their fallopian tubes were either missing or blocked. So the men were not recruited on the basis of their own infertility. “[This] is why we argue that they can be considered a close reflection of the general male population,” Le Moal says.

Using Fécondation *in Vitro* National (FIVNAT), France’s registry of fertility clinic data, the researchers identified 26,609 male partners of women attending 126 clinics all over France between 1989 and 2005. The study relied on data from each couple’s first attempt at assisted reproduction, during which the men, aged 18–70, provided fresh ejaculate. Data on the sperm concentration, morphology, and motility for each sample were extracted for statistical analysis. The investigators computed mean sperm data for each year and adjusted the means to represent an average 35-year-old man.

The men’s average age increased from 34.2 to 35.9 over the 17-year study period. During that time the investigators saw an average 32.2% reduction in mean sperm concentration—an average of 1.9% per year—and the projected concentration for a 35-year-old man fell from an average 73.6 million/mL to an average 49.9 million/mL.^1^ Research published in 2002 suggested it could take longer to achieve pregnancy with a sperm concentration of less than 55 million/mL,^2^ but this threshold has not been established as a clinical indicator, nor has it been replicated by other studies. According to the World Health Organization, sperm concentrations of 15–20 million/mL are considered normal; below that level, the inability to impregnate a partner is far more likely.^3^

The percentage of sperm cells with normal morphology also decreased, from an average 60.9% per sample to an average 52.8% per sample over the same period, or 1.3% per year. (At least part, but probably not all, of this decline can be explained by changes in accuracy of measuring abnormal sperm.) Motility bucked the downward trend, with the percentage of motile sperm in each sample rising from an average 49.5% in 1989 to an average 53.6% in 2005.

The findings support evidence for falling sperm counts reported in earlier studies in France.^4^ Declines have also been reported in smaller studies from countries as far-flung as Israel,^5^ India,^6^ New Zealand,^7^ Tunisia,^8^ and Finland^9^ over the last 10 years. But why should this be happening?

“Sperm counts were higher years ago, so what have been the big changes?” asks professor Richard Sharpe of the MRC Centre for Reproductive Health, University of Edinburgh, UK. “Most attention has focused on increased exposure to environmental endocrine disruptors, but supporting evidence for their effects on sperm count in men is rather weak. The biggest change has been to our diets: We now eat too much, and of the wrong foods.”

Sharpe cites one study showing that men who ate the most saturated fat in their diets had 43% lower sperm counts than men who ate the least.^10^ Another study reported diminished reproductive health in the offspring and grand-offspring of male mice fed a high-fat diet.^11^ “So maybe the diet of our fathers is to blame for low sperm counts in young men,” Sharpe says. “The truth is that we are still speculating about the causes, but it appears certain that it is changes in our modern environment, whether this be chemical exposures or our changing lifestyle or both.”

“Whatever the cause, these results show there is a public health problem that we must address,” says Le Moal. “We need to start research into what is causing this and what we can do about it, and we need to do it now.”
